# Occupational and behavioral factors associated with fatigue among long-distance truck drivers on the Kenya–Uganda transport corridor

**DOI:** 10.3389/fpubh.2026.1831315

**Published:** 2026-07-27

**Authors:** Nelson Kilimo, Kellen Karimi, Olipher Makwaga, Verena Struckmann

**Affiliations:** 1 Department of Public and Global Health, Faculty of Health Sciences, University of Nairobi, Nairobi, Kenya; 2 Center for Infectious and Parasitic Diseases Control Research (CIPDCR), Kenya Medical Research Institute (KEMRI), Busia, Kenya; 3 Department of Health Care Management, Technical University of Berlin, Berlin, Germany

**Keywords:** cross-sectional study, driving hours, East Africa, occupational fatigue, road safety, substance use, truck drivers

## Abstract

**Objectives:**

This study aimed to identify occupational and behavioral factors associated with fatigue among long-distance truck drivers along the Kenya–Uganda transport corridor. A secondary objective was to assess the robustness of identified associations by applying LASSO penalized regression as a sensitivity check.

**Methods:**

A cross-sectional analytical study was conducted among 207 long-distance truck drivers at the Busia and Malaba border crossings. Fatigue was assessed using the Chalder Fatigue Questionnaire (CFQ-11) with binary scoring. Missing data were addressed using multiple imputation by chained equations (MICE; *m* = 20). Factors associated with fatigue were examined using multivariable logistic regression with predictors selected on theoretical grounds from the occupational fatigue literature. LASSO logistic regression was applied as a secondary analysis to confirm that associations were stable under coefficient penalization. Firth penalized logistic regression was conducted as a sensitivity analysis to evaluate the stability of findings.

**Results:**

The prevalence of occupational fatigue was 52.2% (95% *CI*: 44.8–58.6%). Substance use (adjusted *OR* = 8.64; 95% *CI*: 4.30–17.33; *p* < 0.001) and longer daily driving hours (adjusted OR = 1.23 per additional hour; 95% *CI*: 1.10–1.37; *p* < 0.001) were independently associated with fatigue. Both associations were retained under LASSO penalization, supporting their robustness.

**Conclusions:**

Occupational fatigue affects a substantial proportion of long-distance truck drivers and is strongly associated with substance use and extended daily driving hours. Public health and transport-sector interventions should prioritize fatigue prevention through substance-use reduction strategies, driver wellness programmes, and enforcement of regulations limiting excessive driving hours to improve driver health and road safety.

## 1 Introduction

Driver fatigue is a critical global public health and occupational safety concern, particularly within the transport and logistics sectors (1–3). Fatigue impairs vigilance, reaction time, and decision-making capacities essential to safe driving. Fatigue-related crashes are approximately 50% more likely to result in death or severe injury, because drivers who fall asleep typically fail to brake before impact (4, 5).

Occupational fatigue a persistent state of physical or mental exhaustion that reduces a worker's capacity to perform safely arises from prolonged working hours, irregular rest periods, monotonous tasks, and extended wakefulness (6). In the transport industry, physiological contributors include sleep deprivation and circadian rhythm disruption; psychological contributors include high time pressure, social isolation, and limited autonomy (7).

Globally, low- and middle-income countries bear a disproportionate burden of road traffic injuries, accounting for nearly 93% of global road deaths despite having approximately 60% of the world's vehicles (8). In East Africa, the Northern Corridor linking the Port of Mombasa to Uganda and other landlocked countries is a vital freight artery. Drivers face long distances, prolonged border waiting times, variable road conditions, and intense delivery pressure. Despite this occupational burden, evidence on fatigue prevalence and determinants among East African truck drivers remains limited.

Previous studies have identified prolonged driving, inadequate sleep, stimulant use, financial pressure, and limited organizational support as correlates of fatigue; however, most relied on conventional regression methods. Penalized approaches such as LASSO provide an opportunity to examine correlated occupational exposures while reducing overfitting, though results from modestly sized cross-sectional datasets must be interpreted cautiously.

The primary aim of this study was to identify occupational and behavioral factors associated with fatigue among long-distance truck drivers along the Kenya–Uganda transport corridor. A secondary aim was to examine whether identified associations were robust under coefficient penalization, using LASSO as a confirmatory sensitivity analysis. This study is explicitly positioned as an explanatory cross-sectional investigation; no prediction tool is developed or validated.

## 2 Methods

This study was reported in accordance with the Strengthening the Reporting of Observational Studies in Epidemiology (STROBE) statement for cross-sectional studies (26). The STROBE checklist was completed and is provided as a Supplementary file.

### 2.1 Study design and setting

A cross-sectional analytical study was conducted among long-distance truck drivers at the Busia and Malaba border crossing points along the Kenya–Uganda transport corridor. These sites were selected because of their high volume of regional freight movement and prolonged transit times, which provided a naturalistic setting for capturing occupational exposure to fatigue. The customs clearance period of approximately 25 min per vehicle offered a structured and logistically feasible opportunity for recruitment. Systematic random sampling was applied at fixed border crossing points to reduce selection bias within the accessible population; however, it was recognized that drivers sampled at border posts may not fully represent the broader long-distance truck driver population, as those present during clearance may differ in work patterns, experience, compliance behavior, or health status, introducing a potential selection bias analogous to the healthy worker effect.

### 2.2 Study population, eligibility, and sampling

Eligible participants were aged 28 years or older, held valid National Transport and Safety Authority (NTSA) commercial heavy vehicle driving licenses, and had a minimum of 4 years of long-distance truck driving experience. The lower age threshold was selected to align with the Kenyan commercial driver licensing framework, under which drivers seeking authorization to operate articulated heavy trucks and trailers (Class CE) must be at least 28 years old and possess a minimum of 4 years of prior experience operating Class C heavy vehicles. Similarly, drivers operating Class C medium and heavy trucks are required to be at least 24 years old and have a minimum of 2 years of experience driving Class C1 vehicles. These requirements ensured that enrolled participants had substantial occupational exposure to long-distance freight transport and its associated fatigue-related risk factors before enrolment. Drivers currently using medications known to substantially alter sleep architecture or psychomotor alertness were excluded.

Systematic random sampling was applied, the first eligible truck driver encountered during each recruitment period was enrolled, and every fourth subsequent eligible driver was approached until the target sample size was reached. Across both border points, approximately 1,400 trucks transited the corridor during the recruitment period, of whom an estimated 520 drivers were approached and screened for eligibility, yielding 207 enrolled participants after exclusions.

Sample size was estimated using a two-proportion comparison framework (9), assuming 95% confidence, 80% power, and an odds ratio of 2.0 for the primary exposure–outcome association, yielding *n* = 207. This approach is appropriate for the analytical objective of estimating associations between exposures and a binary outcome. The events-per-variable (EPV) ratio for the prespecified four-predictor multivariable model was approximately 27 (108 events/4 predictors), comfortably exceeding the commonly recommended threshold of 10 and supporting the stability of model estimates. For the two-predictor model confirmed by LASSO, the EPV was approximately 54 (108 events/2 predictors), providing additional support for the precision of those estimates.

### 2.3 Outcome measurement

Occupational fatigue was assessed using the Chalder Fatigue Questionnaire (CFQ-11), a validated 11-item instrument measuring physical and mental fatigue dimensions (10, 11). Responses were recoded using the binary scoring approach; a total score of 4 or above defined the fatigued category.

### 2.4 Candidate predictors and operational definitions

Possible risk factors were selected on theoretical grounds from the occupational fatigue literature, not on univariable statistical significance in the current dataset. The four prespecified predictors were age, years of driving experience, average daily driving hours, and substance use while driving. Age and driving experience were included because cumulative physiological wear and irregular schedules associated with long-distance driving have been consistently linked to fatigue in occupational cohorts (27, 28). Daily driving hours were included because exceeding regulated driving-time thresholds is among the most consistently reported proximal determinants of fatigue in commercial drivers (29). Substance use was included because stimulant use to counteract sleepiness is widely documented in sub-Saharan African transport contexts (30, 31).

Substance use was defined as self-reported use of any stimulant including caffeine preparations, khat (miraa), or alcohol to maintain alertness while driving, and was captured as a binary variable. This definition is reported explicitly to support replicability and assessment of construct validity.

### 2.5 Missing data assessment and handling

Missing data patterns were examined prior to any modeling. The variables with the highest rates of missingness were employment type (*n* = 28, 13.5%), gender (*n* = 8, 3.9%), and marital status (*n* = 8, 3.9%); all other variables had minimal missingness (< 3%). Given that 13.5% missingness in employment type could introduce bias under complete-case analysis, multiple imputation by chained equations (MICE) was performed using the *mice* package in R (12). Twenty imputed datasets were generated (*m* = 20). The imputation method was variable-specific: predictive mean matching for continuous variables, logistic regression for binary variables, and polytomous logistic regression for nominal variables. The fatigue outcome was included as a predictor in the imputation model but was not itself imputed. Imputation model convergence was assessed using trace plots. Estimates were pooled across imputed datasets using Rubin's rules (13). A sensitivity analysis compared complete-case and imputed estimates to assess the influence of the imputation strategy.

### 2.6 Statistical analysis

All analyses were conducted in R version 4.3.2 (14), with statistical significance assessed at α = 0.05 (two-sided).

The primary inferential approach was multivariable logistic regression, chosen because the objective was to estimate adjusted associations between prespecified occupational and behavioral predictors and the binary fatigue outcome while accounting for potential confounding. Adjusted odds ratios (aORs) and 95% confidence intervals (CIs) were pooled across imputed datasets using Rubin's rules. Multicollinearity among predictors was assessed using variance inflation factors (VIF) prior to model fitting; no variable exceeded the conventional threshold of *VIF* > 5. No univariable significance threshold was applied to restrict predictor entry; all four prespecified variables entered the multivariable model regardless of their bivariate *p*-values. Bivariate statistics in Table 1 are descriptive only and did not influence variable selection.

**Table 1:** Sociodemographic and occupational characteristics of study participants, stratified by fatigue status.

Characteristic	Overall *N* = 207^a^	No *N* = 99^a^	Yes *N* = 108^a^	*p*-value^b^
Age (years)	41.1 (7.3)	42.1 (7.3)	40.3 (7.2)	0.045
Driving experience (years)	14.0 (6.6)	14.8 (6.9)	13.3 (6.2)	0.092
Substance use while driving	100 (48%)	20 (20%)	80 (74%)	< 0.001
Daily driving hours	12.7 (3.6)	11.2 (3.2)	14.1 (3.3)	< 0.001
Prior fatigue-related accident	118 (57%)	16 (16%)	102 (94%)	< 0.001
Gender^c^				
Male	199 (100%)	94 (100%)	105 (100%)	
Education level				< 0.001
Primary	28 (14%)	22 (22%)	6 (5.6%)	
Secondary	127 (61%)	42 (42%)	85 (79%)	
Tertiary	52 (25%)	35 (35%)	17 (16%)	
Marital status				0.007
Married	199 (96%)	99 (100%)	100 (93%)	
Single	8 (3.9%)	0 (0%)	8 (7.4%)	
Employment type				0.7
Full-time	202 (98%)	96 (97%)	106 (98%)	
Contract/other	5 (2.4%)	3 (3.0%)	2 (1.9%)	
Drives >4 h without break	32 (15%)	32 (32%)	0 (0%)	< 0.001
Work-rest record keeping	169 (82%)	67 (68%)	102 (94%)	< 0.001

^a^Mean (SD); *n* (%).

^b^Wilcoxon rank sum test; Pearson's Chi-squared test; NA, Fisher's exact test.

^c^Gender data available for 199 of 207 participants (8 missing; 3.9%); all 199 with gender data were male.

Standard multivariable logistic regression estimates associations within a fixed predictor set but does not provide a data-driven assessment of which variables retain independent signal when coefficients are penalized for redundancy. To assess whether the associations identified in the primary model were robust to such penalization, LASSO logistic regression was applied as a secondary analysis using the *glmnet* package (15). For reproducibility, a random seed of 123 was set prior to performing multiple imputation, and a separate random seed of 456 was set prior to LASSO cross-validation to ensure reproducible fold assignment. LASSO applies an L_1_ penalty that shrinks weak or redundant coefficients toward zero; predictors retained despite this penalization can be regarded as more consistently associated with the outcome. This analysis was used solely to confirm the robustness of associations identified in the primary model, not to develop a prediction tool or select a final variable set. Continuous predictors were standardized prior to penalization to ensure comparability of shrinkage. The tuning parameter (λ) was selected using 10-fold cross-validation with the λ_1se_ criterion to favor parsimony. LASSO-derived coefficients were not interpreted as inferential estimates.

The area under the receiver operating characteristic curve (AUC) was estimated as a descriptive measure of the primary model's ability to discriminate between fatigued and non-fatigued drivers. No formal predictive validation was performed, consistent with the explanatory intent of the study.

Firth penalized logistic regression was applied to evaluate the stability of parameter estimates under potential sparse-data conditions (16, 17). Complete-case estimates were also compared with multiply imputed estimates to assess the robustness of findings to the missing-data strategy.

### 2.7 Conceptual framework

**Figure 1:**
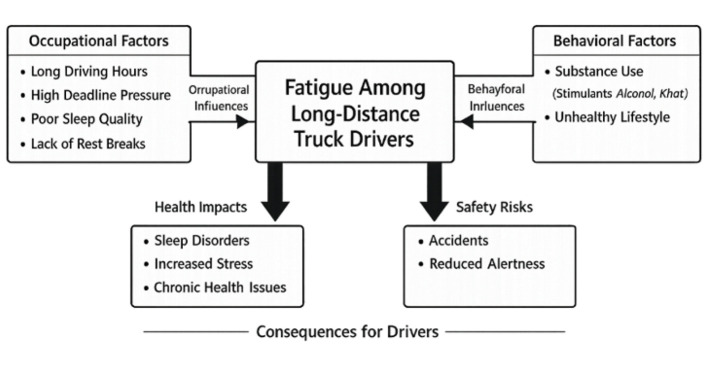
Conceptual framework adopted to understand fatigue in long-distance truck drivers.

The analytical approach in this study was guided by a direct-association framework linking occupational and behavioral exposures to fatigue outcomes, without positing mediation or latent variable structures as summarized in the conceptual framework (Figure 1). The conceptual diagram presented in the Supplementary materials reflects this direct-association model; any pathway representations from prior versions of the manuscript that implied mediation or latent constructs have been revised accordingly, in response to reviewer feedback.

### 2.8 Ethical considerations

Informed consent was obtained from all participants. Ethical approval was granted by the KNH-UON Ethics and Research Committee (Approval No. P555/07/2024). Research authorization was issued by NACOSTI (Permit No. NACOSTI/P24/414364). Administrative notifications were provided to KENHA, Customs Offices at Busia and Malaba, and Busia County Government.

## 3 Results

### 3.1 Participant characteristics and fatigue prevalence

A total of 207 male participants were enrolled in the study. The mean age was 41.1 years (*SD* 7.3), with the majority aged 40–49 years (44.4%). Most participants were married (91.8%), had attained secondary education (61.8%), and were employed full-time (84.5%). The mean driving experience was 14.0 years (*SD* 6.6), and average daily driving hours were 12.7 (*SD* 3.6). The pooled prevalence of occupational fatigue was 52.2% (108 of 207; 95% *CI*: 44.8–58.6%). Notable occupational characteristics included substance use while driving (48%), prior fatigue-related accidents (57%), driving without breaks for more than 4 h (15%), and poor work–rest record-keeping (18%).

Participant characteristics stratified by fatigue status are presented in Table 1. Significant between-group differences were observed for age, education level, marital status, daily driving hours, substance use, prior fatigue-related accidents, driving without breaks, and work–rest record keeping (all *p* < 0.05). Driving experience and employment type did not differ significantly between groups.

### 3.2 LASSO variable selection

**Figure 2:**
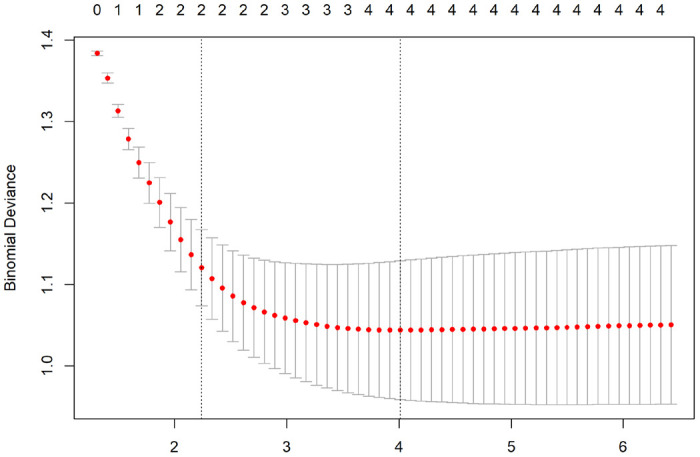
Cross-validation curve for LASSO logistic regression.

**Figure 3:**
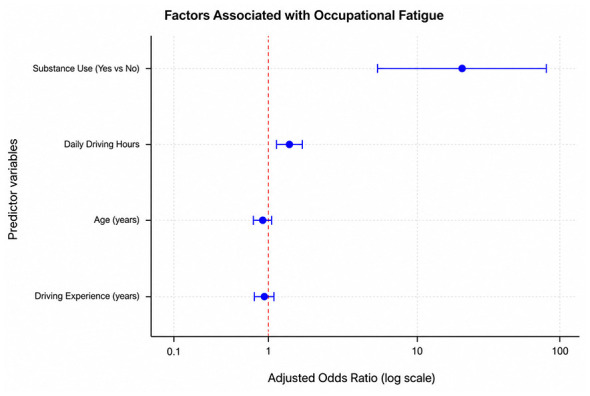
Forest plot of factors associated with occupational fatigue.

**Figure 4:**
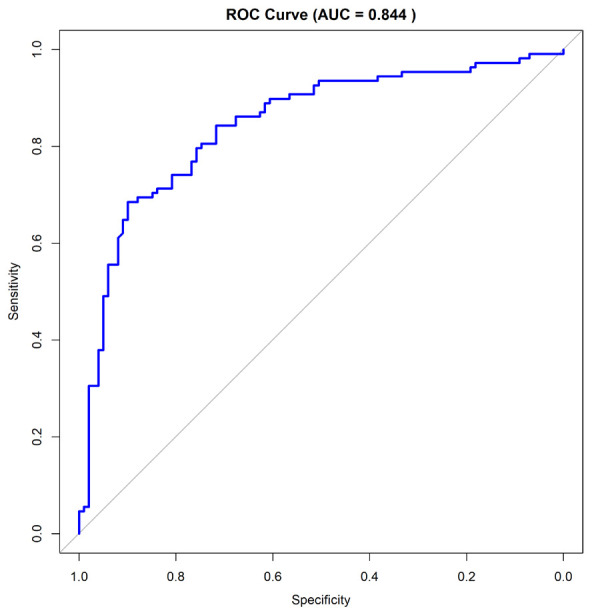
Receiver operating characteristic (ROC) curve for the primary multivariable logistic regression model (reported as a supplementary robustness check).

LASSO logistic regression applied to the prespecified predictor set retained two variables at the λ_1s_E penalty: substance use and daily driving hours. The corresponding cross-validation error curve is shown in Figure 2 (LASSO binomial deviance plot), and the coefficient shrinkage paths are presented in Figure 3. To aid interpretation, the adjusted odds ratios for all candidate predictors are displayed in Figure 4 as a forest plot, highlighting the relative strength of associations.

The retained LASSO coefficients were: substance use (β = 1.226) and daily driving hours (β = 0.054); the intercept was −1.176. The reduction to two predictors reflects the regularizing effect of LASSO given the sample size. This does not imply that other candidate variables are causally unimportant, but rather that they contributed minimal marginal discrimination in this dataset.

The forest plot displays adjusted odds ratios from the multivariable logistic regression. Substance use was strongly associated with fatigue (adjusted *OR* = 8.64; 95% *CI*: 4.30–17.33), while longer daily driving hours also showed a significant dose–response effect (adjusted *OR* = 1.23 per additional hour; 95% *CI*: 1.10–1.37). In contrast, age and driving experience were not independently associated, with confidence intervals crossing the null line. These findings highlight behavioral and occupational exposures rather than demographic factors as the primary drivers of fatigue risk among long-distance truck drivers. Both substances use and driving hours remained strong risk factors for occupational fatigue under LASSO penalization, underscoring the robustness of these associations.

### 3.3 Multivariable logistic regression

The pooled multivariable logistic regression included all four prespecified predictors (Table 2). This full-model approach was adopted to estimate adjusted associations for each variable while accounting for the others, consistent with the explanatory intent of the study. Substance use was the strongest predictor (adjusted *OR* = 8.64; 95% *CI*: 4.30–17.33; *p* < 0.001), indicating that substance-using drivers had over eight-fold higher odds of fatigue compared to non-users. Daily driving hours also significantly predicted fatigue (adjusted *OR* = 1.23 per additional hour; 95% *CI*: 1.10–1.37; *p* < 0.001), suggesting that each additional hour of daily driving was associated with a 23% increase in the odds of fatigue. Both predictors were subsequently confirmed by LASSO penalization as retaining meaningful signal under coefficient shrinkage.

**Table 2:** Multivariable logistic regression results.

Term	Estimate	2.5 %	97.5 %	*p*-value
Substance use (yes vs. no)	8.635	4.302	17.332	0.000
Daily driving hours	1.228	1.097	1.374	0.000
Age (years)	0.966	0.910	1.026	0.258
Driving experience (years)	0.987	0.924	1.054	0.700

Age and driving experience were not independently associated with fatigue (*p* = 0.258 and *p* = 0.700, respectively). The confidence intervals for substance use are consistent with the distributional properties of a binary predictor in a dataset of this size and indicate appropriate uncertainty around point estimates. Firth penalized logistic regression sensitivity analysis confirmed the same direction of effect for both predictors. These findings are interpreted as exploratory associations requiring replication in larger independent samples.

### 3.4 Model fit and discrimination metrics

As a supplementary robustness check requested by reviewers, model discrimination and calibration were assessed for the primary multivariable logistic regression. The apparent AUC was 0.844; bootstrap correction yielded an AUC of 0.831 (optimism = 0.013), indicating stable performance with minimal overfitting. These metrics are reported solely to address reviewer concerns and do not reposition this work as a predictive modeling study. Calibration was adequate, as indicated by a non-significant Hosmer–Lemeshow test (*p* = 0.243) and a Brier score of 0.159. The ROC curve is shown in Figure 4.

Contextual model metrics: *n* = 207, 20 imputed datasets, pooled fatigue prevalence of 52.2%, *EPV* = 27 for the four-predictor model and *EPV* = 54 for the two-predictor LASSO-confirmed model. The AUC and calibration statistics are interpreted as indicators of model fit and analytical robustness in this cross-sectional sample, not as evidence that the model is ready for clinical or operational use. No external validation has been performed.

## 4 Discussion

### 4.1 Principal findings

This study identified substance use and daily driving hours as the two variables most strongly and independently associated with occupational fatigue among long-distance truck drivers along the Kenya–Uganda transport corridor. A pooled fatigue prevalence of 52.2% is elevated relative to global estimates of 25–45% (18), consistent with the particularly demanding operational conditions of this corridor, including prolonged border transit times and intense commercial scheduling pressure.

This is an explanatory cross-sectional study; the primary goal is to identify occupational and behavioral correlates of fatigue, not to develop a clinical prediction tool. Wide confidence intervals around the substance use OR reflect the binary distributional properties of this predictor in this sample and warrant cautious interpretation. The primary scientific contribution of this work is identification of the occupational correlates of fatigue, not development of a deployable prediction tool.

### 4.2 Daily driving hours as the dominant occupational exposure

The association between daily driving hours and fatigue (*aOR* = 1.23 per additional hour) is consistent with prior evidence from LMICs and high-income settings alike (3, 19). In the Northern Corridor context, border waiting times compound already demanding schedules; drivers may accumulate many hours behind the wheel before and after clearance without adequate rest. The continuous dose–response relationship suggested by the OR per driving hour is particularly noteworthy from a regulatory standpoint.

This finding aligns with the job demands–resources model (20), which posits that high demands in the absence of adequate resources drive worker exhaustion. The significance of daily driving hours in the model suggests that enforcement of hours-of-service regulations and driver welfare policies represent high-leverage targets. Deadline pressure, while not retained as an independent model predictor, likely operates as a structural upstream driver of excessive driving hours and remains an important policy target.

### 4.3 Substance use as a behavioral coping response

Substance use was independently associated with over eight-fold increased odds of fatigue. Consistent with evidence from Brazil (21) and other contexts, stimulant use appears to function as a short-term response to wakefulness demands, while disrupting sleep architecture and accumulating sleep debt, thereby paradoxically exacerbating fatigue over time (22). The independent significance of substance use after adjustment for daily driving hours suggests a partially direct pathway to fatigue, beyond its role as a compensatory alertness strategy in the context of extended driving shifts.

### 4.4 Comparison with global literature

Chen and Zhang (23) reported 24.8% fatigue prevalence among Chinese long-distance truck drivers, with higher rates under tight schedules and extended driving hours, supporting the daily driving hours finding. Studies from Europe and Latin America similarly identify long hours, night driving, and stimulant use as key correlates (21, 24, 25). Effect sizes in prior studies are generally more modest than those observed here, which may reflect differences in regulatory enforcement, infrastructure, border dynamics, and cultural contexts of stimulant use. Direct comparisons are constrained by methodological heterogeneity across studies in fatigue instruments, predictor operationalization, and analytical approaches.

### 4.5 Non-significant variables

Several variables examined in descriptive analyses including employer support, ILO regulatory knowledge, and work–rest record-keeping were not retained in the final model. Per reviewer recommendations, the discussion focuses on the two retained predictors to avoid over-interpretation of variables absent from the model. The non-retention of these variables likely reflects the statistical dominance of substance use and daily driving hours in this sample size context, rather than their theoretical unimportance. Larger studies with greater power are needed to evaluate their independent contributions.

### 4.6 Strengths and limitations

Strengths of this study include use of a validated fatigue instrument, systematic sampling at high-volume border crossings, rigorous missing data handling via MICE, and a coherent analytical framework combining explanatory logistic regression with exploratory LASSO penalization, with reporting following STROBE guidelines for cross-sectional observational studies.

Important limitations must be acknowledged. The cross-sectional design precludes causal inference and cannot exclude reverse causality. Self-reported data introduce social desirability and recall bias. The all-male sample from two border points limits generalizability to female drivers and other corridors. The prespecified four-predictor multivariable model achieved an EPV of approximately 27 (108 events/4 predictors), meeting accepted thresholds for stable logistic regression; the LASSO-confirmed two-predictor model achieved an EPV of approximately 54. Nonetheless, wide confidence intervals around the substance use OR reflect the binary distribution of this predictor and warrant cautious interpretation. Systematic random sampling at border crossings may introduce selection bias if waiting drivers differ from those in transit; specifically, drivers with severe fatigue may have stopped at earlier points, introducing healthy worker bias. The LASSO tuning parameter was selected using cross-validation on a single imputed dataset rather than across all imputations, which is a pragmatic but acknowledged simplification. External validation in an independent sample is required before predictive estimates can be generalized.

### 4.7 Implications for policy and practice

For policymakers and regulators: enforcement of driving-hour regulations must move beyond paper compliance to demonstrated outcomes. The Kenya Road Safety Policy's recommendations for structured rest breaks and daily driving limits provide an appropriate framework, but require resourcing for compliance monitoring.

For employers: scheduling practices that accommodate realistic transit times including border delays would reduce the structural driver of fatigue. Substance use interventions that adopt a health-oriented, non-punitive approach are likely more effective than deterrence alone.

For future research: longitudinal studies are needed to establish causal sequences and evaluate intervention effectiveness. Studies with larger and more geographically diverse samples, including female drivers, would improve generalizability and statistical precision.

### 4.8 Conclusion

Occupational fatigue is highly prevalent among long-distance truck drivers along the Kenya–Uganda transport corridor and is most strongly associated with substance use and cumulative daily driving hours. These explanatory findings from a cross-sectional study suggest that fatigue in this population is driven by modifiable behavioral and occupational exposures. Effective interventions must prioritize hours-of-service enforcement and behavioral health support for substance use, alongside upstream scheduling reform. Future longitudinal studies with larger and more diverse samples are needed to establish causal sequences and support generalization of these findings.

## Data Availability

The raw data supporting the conclusions of this article will be made available by the authors, without undue reservation.
